# Multimodal DTI-ALPS and hippocampal microstructural signatures unveil stage-specific pathways in Alzheimer’s disease progression

**DOI:** 10.3389/fnagi.2025.1609793

**Published:** 2025-07-28

**Authors:** Peng Yu, Lu Shen, Lijun Tang

**Affiliations:** ^1^Department of Radiology, Taixing People's Hospital, Taixing, China; ^2^Department of Nuclear Medicine, The First Affiliated Hospital of Nanjing Medical University, Jiangsu Province Hospital, Nanjing, China; ^3^School of Medical Imaging, Nanjing Medical University, Nanjing, China

**Keywords:** Alzheimer’s disease, mild cognitive impairment, DTI-ALPS index, hippocampal microstructure, fractional anisotropy, mean diffusivity, CSF biomarkers

## Abstract

**Objective:**

Develop a multimodal biomarker framework integrating DTI-ALPS (Diffusion Tensor Imaging along the Perivascular Space), hippocampal diffusivity, and CSF profiles for staging Alzheimer’s disease (AD) progression across the HC → MCI → AD continuum.

**Methods:**

Cross-sectional analysis of 60 age-matched participants [18 healthy controls (HC), 20 with mild cognitive impairment (MCI), and 22 with Alzheimer’s disease (AD)] combining 3 T MRI-derived biomarkers (bilateral hippocampal fractional anisotropy (FA) and mean diffusivity (MD), and DTI-ALPS). Cerebrospinal fluid (CSF) analysis (Aβ42, p-tau181, t-tau), and cognitive assessments (MMSE, MoCA). Statistical analyses included ANOVA with Bonferroni correction, Pearson correlations, and ROC curve evaluation for disease classification.

**Results:**

DTI-ALPS exhibited a progressive decline (HC: 1.31 ± 0.12 → MCI: 1.26 ± 0.09 → AD: 0.87 ± 0.19; *p* < 0.001 for AD vs. HC/MCI). Bilateral FA reductions plateaued in MCI (left: 0.57 ± 0.11 vs. HC: 0.82 ± 0.07, *p* < 0.001; right: 0.57 ± 0.11 vs. HC: 0.80 ± 0.07, *p* < 0.001) without further progression at the AD stage. MD showed a right-lateralized progression (HC → MCI → AD: left 0.53 → 0.74 → 0.78, right 0.51 → 0.71 → 0.77; *p* < 0.001), with a significant increase only in right MD from MCI to AD (*p* = 0.014). CSF biomarkers revealed a hierarchical depletion of Aβ42 (AD: 370.7 ± 145.9 vs. HC: 910.8 ± 191.5 pg./mL, *p* < 0.001) and accumulation of tau (t-tau: AD>MCI > HC, *p* < 0.001). Receiver operating characteristic (ROC) analysis identified right hippocampal MD and t-tau as optimal classifiers for AD.

**Conclusion:**

The framework reveals distinct biomarker trajectories: DTI-ALPS distinguishes symptomatic AD from preclinical stages, while right hippocampal MD progression reflects tau-mediated neurodegeneration. Early FA reductions in MCI combined with CSF profiles suggest a hierarchical staging model: amyloid-associated perivascular dysfunction is associated with asymmetric tau-driven hippocampal degeneration. This multimodal approach provides clinically actionable biomarkers for AD progression monitoring.

## 1 Introduction

Mild cognitive impairment (MCI), defined as cognitive decline beyond age-related norms in the absence of functional disability (Duff et al., 2010), has emerged as a critical therapeutic window for preventing dementia progression. Large-scale epidemiological studies reveal a prevalence of 15–20% in adults aged 60 years and older, with an annual conversion rate to Alzheimer’s disease (AD) of 8–15% (Hansson et al., 2018). This prodromal state underscores the urgency to identify clinically actionable biomarkers predicting AD conversion.

**Figure 1:**
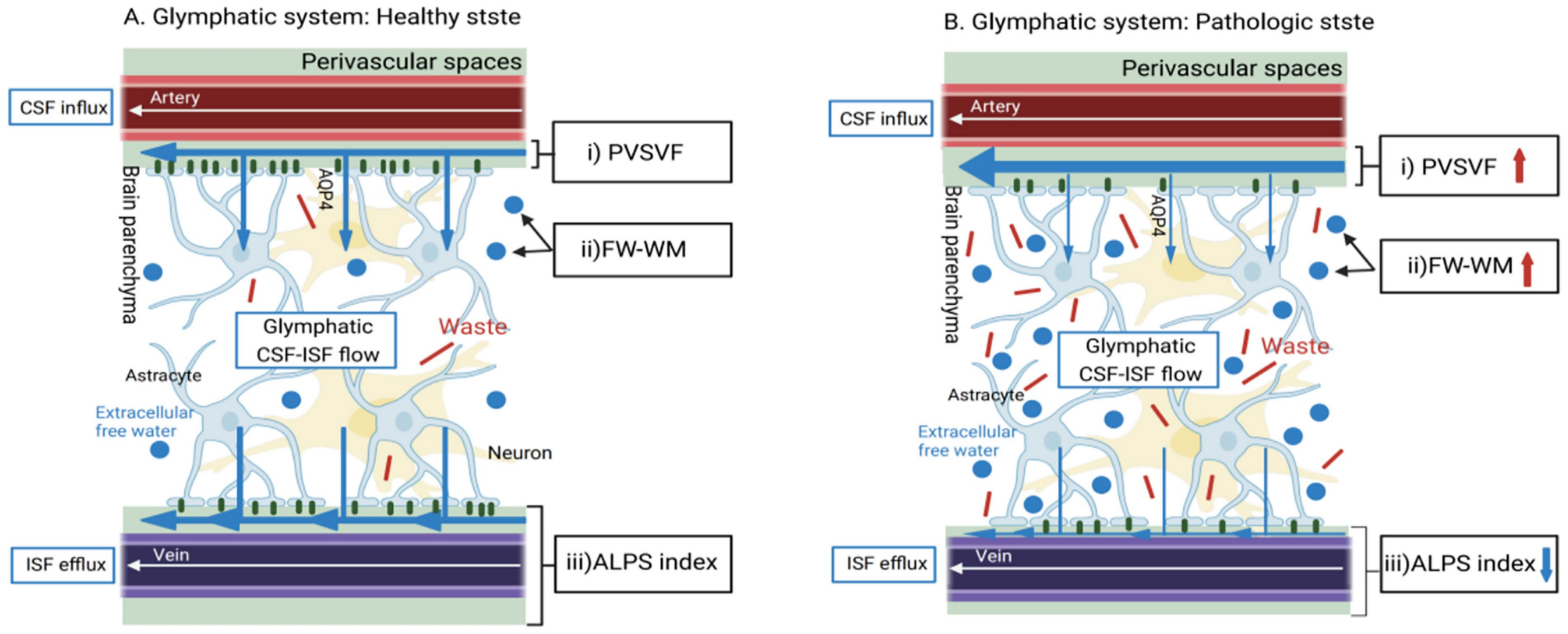
Basic concepts of MRI methods for the indirect, non-invasive evaluation of different aspects of the cerebral perivascular structural integrity in healthy and pathological conditions. **(A)** In healthy states, CSF would enter the brain parenchyma interstitial space through the foot processes of microglia from the perivascular space along the perivascular spaces (PVS) surrounding the arteries (Pasternak et al., 2009). It would then mix with the interstitial fluid and waste products of the brain parenchyma (Zhou et al., 2025). Illustrates the normal flow of CSF through the perivascular spaces and its exchange with ISF. This would produce waste products such as t-tau, which would then be excreted from the brain via the peripheral venous drainage pathway, assessing perivascular structural integrity (indicated by the DTI-ALPS index). **(B)** Glymphatic System in Pathological State: Depicts the impaired glymphatic clearance and the resulting accumulation of waste products, such as amyloid-β, in Alzheimer’s disease. The DTI-ALPS index showed significant reduction (*p* < 0.001), reflecting perivascular system dysfunction (Table 1).

Recent advances have delineated two distinct biomarker axes: (1) Cerebrospinal fluid (CSF) amyloid-tau profiles demonstrating 85–90% diagnostic accuracy in distinguishing MCI-AD converters (Mestre et al., 2018); (2) Diffusion tensor imaging (DTI) revealing microstructural white matter disorganization that longitudinally correlates with cognitive decline (Hasegawa et al., 2024). Significantly, the novel DTI-ALPS (Diffusion Tensor Imaging Along the Perivascular Space) method precisely quantifies perivascular fluid dynamics (Harrison et al., 2020), providing mechanistic insights into glymphatic dysfunction—a well-characterized driver of amyloid accumulation in preclinical models (Figure 1; Sperling et al., 2011).

**Table 1:** Clinical characteristics of the HC, MCI, and AD groups.

	HC *N* = 18	MCI *N* = 20	AD *N* = 22	*p* value			
				HC vs MCI vs AD	HC vs MCI	HC vs AD	MCI vs AD
Sex (Male/Female)	7/11	13/7	11/11	0.279	0.253	0.766	0.6
Age (y)	69.33 ± 12.69	66.9 ± 13.4	68.77 ± 9.9	0.803	0.807	0.988	0.869
Education (y)	10.28 ± 2.85	11.6 ± 3.22	11.32 ± 2.51	0.336	0.337	0.492	0.946
Cognition							
MMSE (N)	28.28 ± 1.13	21.5 ± 3.5	10.1 ± 3.87	**<0.001**	**<0.001**	**<0.001**	**<0.001**
MOCA (N)	28.17 ± 1.3	18.05 ± 4.27	10.55 ± 3.6	**<0.001**	**<0.001**	**<0.001**	**<0.001**
CSF							
Aβ42 (pg/mL)	910.8 ± 191.46	792.9 ± 129.4	370.7 ± 145.87	**<0.001**	0.06	**<0.001**	**<0.001**
p-tau181 (pg/mL)	31.0 ± 8.18	48.8 ± 13.0	86.6 ± 24.0	**<0.001**	**0.006**	**<0.001**	**<0.001**
t-tau (pg/mL)	258.73 ± 82.5	411.7 ± 78.18	539.08 ± 102.95	**<0.001**	**<0.001**	**<0.001**	**<0.001**
MRI							
Left-ALPS	1.3 ± 0.14	1.24 ± 0.11	0.89 ± 0.21	**<0.001**	0.599	**<0.001**	**<0.001**
Right-ALPS	1.32 ± 0.14	1.3 ± 0.1	0.86 ± 0.2	**<0.001**	0.722	**<0.001**	**<0.001**
ALPS mean	1.31 ± 0.12	1.26 ± 0.09	0.87 ± 0.19	**<0.001**	0.608	**<0.001**	**<0.001**
L-hippo FA	0.82 ± 0.07	0.57 ± 0.11	0.57 ± 0.13	**<0.001**	**<0.001**	**<0.001**	0.969
R-hippo FA	0.80 ± 0.07	0.57 ± 0.11	0.58 ± 0.13	**<0.001**	**<0.001**	**<0.001**	0.903
L-hippo MD	0.53 ± 0.07	0.74 ± 0.07	0.784 ± 0.07	**<0.001**	**<0.001**	**<0.001**	0.067
R-hippo MD	0.51 ± 0.06	0.71 ± 0.06	0.77 ± 0.07	**<0.001**	**<0.001**	**<0.001**	**0.014**

Continuous variables are presented as mean ± SD; categorical variables as count (%), unless stated otherwise. Data were compared using ANOVA. Bold values indicate statistically significant differences (*p* < 0.05) among HC, MCI, and AD groups, highlighting the robustness of findings related to Alzheimer’s disease progression. HC, Healthy control group; MCI, Mild Cognitive Impairment; AD, Alzheimer’s disease; MMSE, Mini-Mental State Examination; MoCA, Montreal Cognitive Assessment; CSF, Cerebrospinal fluid; Aβ42, amyloid β peptide 1–42; t-tau, Total tau; p-tau181, Phosphorylated tau181; ALPS, Analysis along the perivascular space; FA, Fractional anisotropy; MD, Mean diffusivity; VOI, Volume of interest.

The hippocampus, an affective and unique memory and learning structure, plays a pivotal role in Alzheimer’s disease progression. Advanced neuroimaging biomarkers, including amyloid and tau depositions, hippocampal subfields volumetry, diffusivity, default mode network activity, and connectivity, have been extensively examined to elucidate hippocampal involvement (Zhou, 2021).

However, three critical knowledge gaps persist: First, 30–40% of MCI patients exhibit discordant biomarker-imaging profiles (Iulita et al., 2023), compromising current diagnostic frameworks. Second, the spatiotemporal relationship between glymphatic impairment (ALPS index) and hippocampal neurodegeneration remains poorly characterized. Third, no prior study has systematically integrated ALPS metrics with CSF biomarkers for AD continuum stratification.

To address these gaps, this study employs a multimodal neuroimaging approach combining DTI-ALPS, hippocampal diffusivity metrics, and CSF proteomic profiling. We hypothesize that: (1) ALPS index reduction specifically marks AD-stage pathology (Zhou et al., 2024); (2) Right-lateralized hippocampal mean diffusivity (MD) progression reflects tau-mediated asymmetric neurodegeneration; (3) Combined ALPS/MD metrics improve diagnostic accuracy beyond single-modality biomarkers.

## 2 Materials and methods

### 2.1 Subjects

In accordance with the ethical guidelines, this study retrospectively enrolled MCI and AD patients who were admitted to The First Affiliated Hospital of Nanjing Medical University from February 2023 to October 2024, All patients underwent standardized neurological, neuropsychological, and MRI/CSF assessments (Zhou, 2021). Alzheimer’s disease (AD) patients met the 2018 NIA-AA core clinical-biological criteria for Alzheimer’s dementia (Jack et al., 2018), MCI participants fulfilled Petersen’s clinical criteria requiring subjective cognitive complaints and objective impairment in 1–2 cognitive domains with preserved daily functioning (Petersen, 2016), Healthy controls (HC) demonstrated no cognitive complaints [Clinical Dementia Rating (CDR) = 0] (Morris, 1993) and education-matched normative scores. MMSE/MoCA cutoffs were education-stratified: Illiterate: MMSE≤17 (AD), ≥17 (MCI), ≥27 (HC); MoCA≥13 (AD), ≥13 (MCI), ≥26 (HC); Primary education: MMSE≤20 (AD), ≥20 (MCI), ≥28 (HC); MoCA≥19 (MCI), ≥26 (HC); Secondary/higher: MMSE≤24 (AD), ≥24 (MCI), ≥28 (HC); MoCA≥24 (MCI), ≥27 (HC).

### 2.2 Technique for examination

We used the GE 3.0 T PET-MR scanner and a 32-channel high-resolution cranial orthogonal coil. We employ high-resolution DTI sequences. The diffusion gradient directions are scanned in 30 directions. Diffusion-weighted images acquired at a b-value of 1,000 s/mm^2^ in each direction.

The specific parameters are as follows: slice thickness is 2.43 mm, slice gap is 3 mm; TR is 3,000 ms, TE is 90 ms; matrix is 128 × 128; FOV is 200 mm × 231 mm × 119 mm; total scanning time is approximately 6 min. The scanning range extends from the base of the skull to the top of the skull.

**Figure 2:**
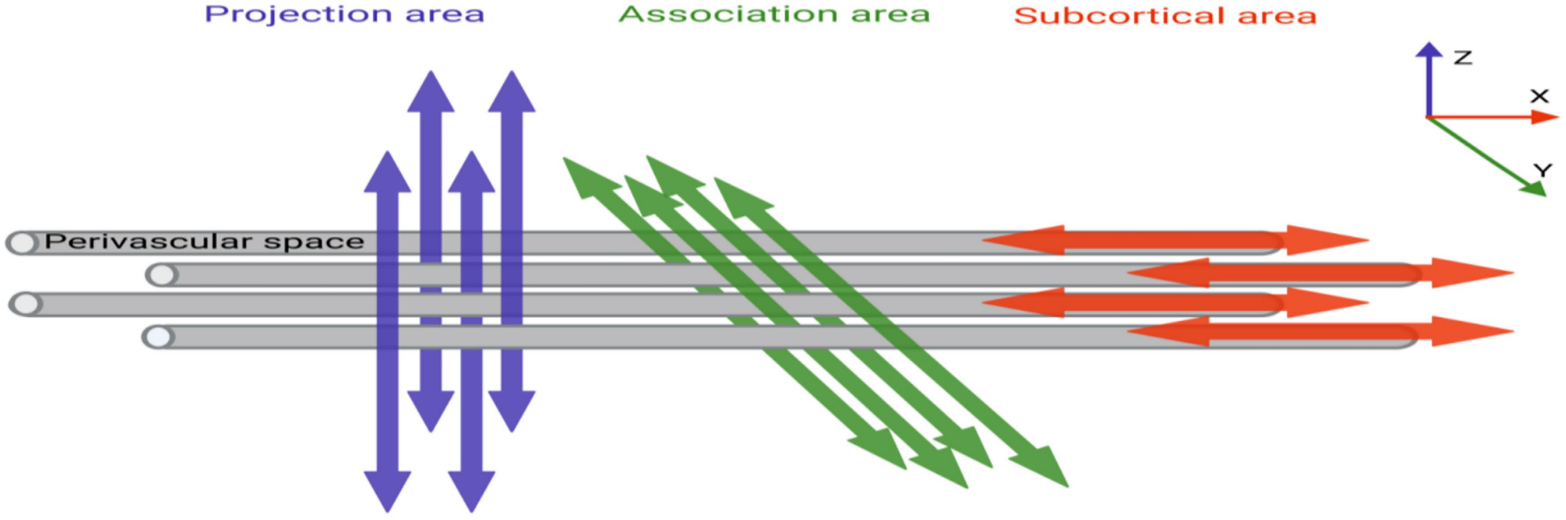
The relationship between perivascular spaces (gray cylinders) and the directions of projection and association fibers.

**Figure 3:**
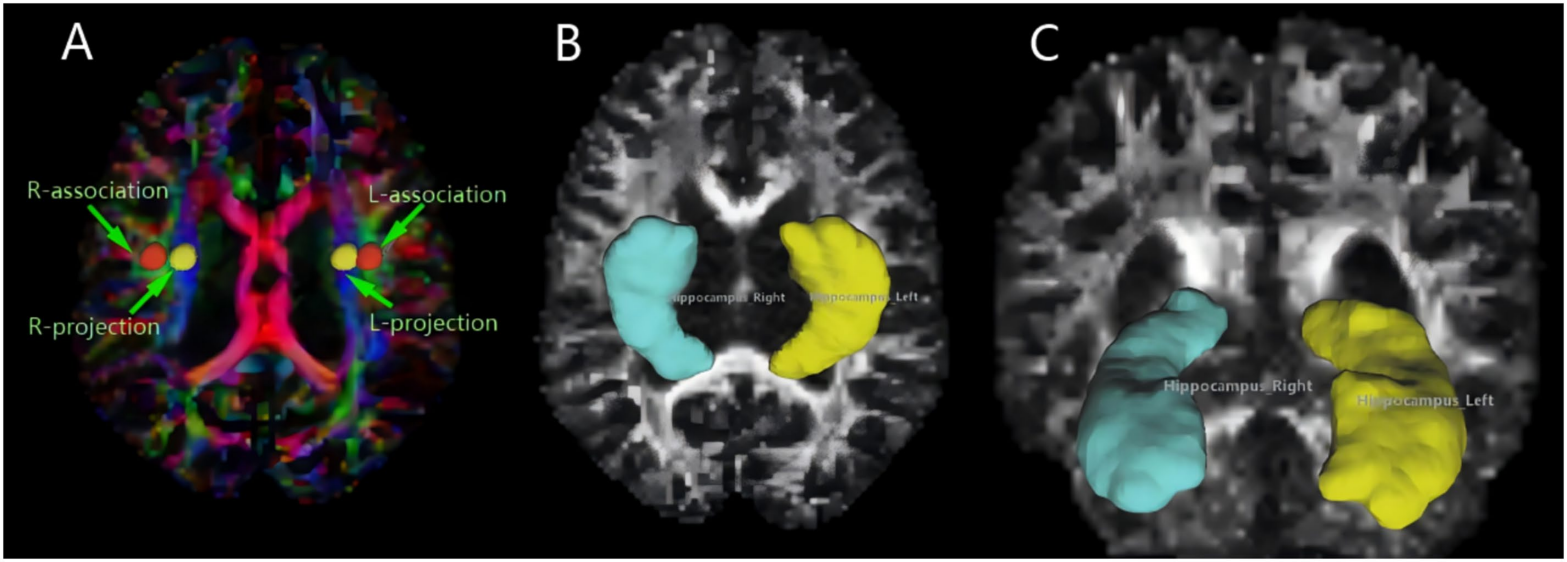
MRI image omics diagram. **(A)** The color-coded FA map illustrates the lateral ventricle body, featuring a 5 mm diameter spherical ROI on both the left and right sides. The ROI for the associated fibers is labeled “projection” (Y-axis, green), and the ROI for the projective fibers is labeled “association” (Z-axis, blue). **(B,C)** Using DSI-studio software, we mapped the volume of the hippocampus, our region of interest. Different brain regions exhibit varying morphologies of the hippocampus.

The DTI-ALPS index quantifies perivascular structural integrity by analyzing diffusion anisotropy along the perivascular spaces (PVS) at the lateral ventricle level. This method leverages the directional dependence of water diffusion in PVS: in healthy states, the X-axis (projection fibers) and Z-axis (association fibers) exhibit higher diffusivity due to alignment with perivascular flow (Figure 2), the 5 mm ROIs in the projection and association regions are aligned along the x-axis in Figure 3, thereby maintaining consistency in the calculation of ALPS values (Figure 3). While the Y-axis (perpendicular to PVS) shows restricted diffusion. The ALPS index reflects the ratio of parallel-to-perpendicular diffusivity, with lower values indicating impaired PVS function (Taoka et al., 2017).

### 2.3 Cerebrospinal fluid (CSF) biomarker analysis

Concentrations of amyloid-*β* 1–42 (Aβ42), phosphorylated tau 181 (p-tau181), and total tau (t-tau) were quantified using commercially available enzyme-linked immunosorbent assay (ELISA) kits. All samples were analyzed in a single batch to minimize variability. To align with recent technological advances and improve diagnostic precision, our analytical approach was validated against fully automated chemiluminescence enzyme immunoassay (CLEIA) platforms, which demonstrate superior analytical performance for t-tau and p-tau181 detection (Arcaro et al., 2022).

### 2.4 Definition of biomarker positivity

Positivity thresholds followed established international criteria (Wang et al., 2024):

Aβ42: Levels <530 pg./mL indicated amyloid pathology (positivity).
p-tau181: Levels >60 pg./mL indicated tau pathology (positivity). This marker significantly enhances differential diagnosis accuracy when combined with Aβ42, particularly in excluding conditions like Creutzfeldt-Jakob disease.
t-tau: Levels >300 pg./mL indicated neurodegeneration (positivity). Notably, the ratio of p-tau181/Aβ42 or t-tau/Aβ42 may provide higher specificity, though single thresholds were used here per consensus guidelines.

### 2.5 Data processing

In Windows PowerShell, the raw DTI image dicom files collected were converted into nii files using the dcm2niix plug-in. Subsequently, the nii files were post-processed in the DSI-studio software, as follows: (1) First, image quality checks were performed, and a mask was set to filter out the background region. This helps improve the reconstruction efficiency and facilitates subsequent visualization. (2) ALPS region of interest (ROI) selection: A circular ROI with a diameter of 5 mm is placed in the projection and association fibers within the lateral ventricle body level (Figure 3A). (3) According to the software’s algorithms and formulas, the DTI-ALPS index is calculated, the formula being: $\mathrm{DTI}-\text{ALPS index}=\frac{\text{mean}(\mathrm{Dxx}-\text{proj},\mathrm{Dxx}-\text{assoc})}{\text{mean}(\mathrm{Dyy}-\text{proj},\mathrm{Dzz}-\text{assoc})}$ Here, Dxx-proj and Dxx-assoc, respectively, represent the diffusion rates of the projection and association fibers along the X-axis; Dyy-proj represents the diffusion rate of the projection fibers along the Y-axis; and Dzz-assoc represents the diffusion rate of the association fibers along the Z-axis. (4) Hippocampus volume of interest (VOI): In the DSI-studio software, the left and right hippocampal regions were manually selected, and the system automatically outlines these regions and calculates the FA and MD values (Figures 3B,C). Hippocampal diffusivity metrics (FA, MD) were calculated using a deterministic fiber tracking algorithm with an FA threshold of 0.2 and angular threshold of 60^o^. Manual segmentation followed the EADC-ADNI harmonized protocol (Duchesne et al., 2015), ensuring anatomical consistency across subjects. To minimize partial volume effects from adjacent CSF, voxels with MD > 1.2 × 10^−**3**^ mm^**2**^/s were excluded.

### 2.6 Statistical analysis

All statistical analyses were conducted using IBM SPSS 26.0 with the following protocol.

#### 2.6.1 Descriptive statistics

Continuous variables (age, cognitive scores, CSF biomarkers, MRI metrics) are reported as mean ± standard deviation. Categorical variables (sex) are expressed as counts and percentages (%), with group differences assessed via chi-square tests.

#### 2.6.2 Group comparisons

Prior to performing the one-way ANOVA, we conducted Shapiro–Wilk normality tests on all continuous variables to verify the assumption of normal distribution. The results confirmed that all variables used in the ANOVA analysis were normally distributed (*p* > 0.05 for all tests), thus justifying the use of parametric statistics. We then evaluated inter-group differences (HC vs. MCI vs. AD) for continuous variables using one-way ANOVA, followed by *post hoc* Bonferroni-corrected pairwise comparisons to control for Type I error. Statistical significance was defined as a two-tailed *p* < 0.05.

#### 2.6.3 Correlation analysis

Pearson’s correlation coefficients (r) were calculated to assess linear relationships between MRI metrics (ALPS index, hippocampal FA/MD) and clinical variables (MMSE, MoCA, CSF biomarkers). Significance thresholds were set at two-tailed *p* < 0.05 without additional multiple comparison correction.

### 2.7 Data visualization

**Figure 4:**
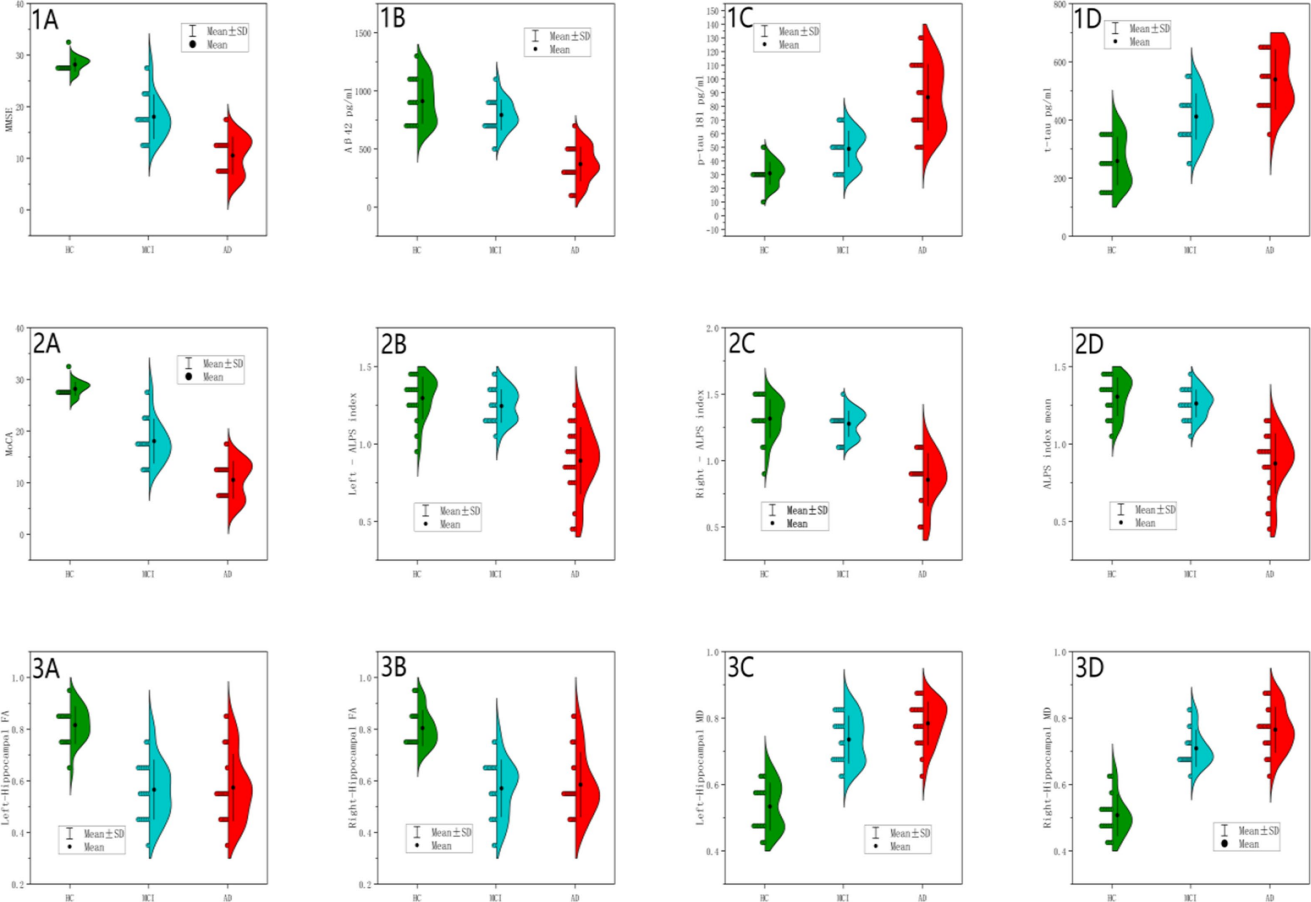
Between-group differences in cognitive scores, CSF biomarkers and MRI measurements. Panels **(1A,2A)** (MMSE/MoCA Scores): HC showed tightly clustered distributions; AD showed severe reduction; MCI demonstrated intermediate values, consistent with global cognitive decline (*p* < 0.001). Panel **(1B)** (CSF Aβ42): HC (blue): Concentrated distribution at higher values with tight distribution, indicating high intra-group homogeneity. MCI (green): Left-shifted distribution with increased dispersion and partial distribution overlap with HC, reflecting Aβ42 levels comparable to HC in some MCI individuals. AD (red): Marked leftward shift with narrow density and minimal overlap, demonstrating universal Aβ42 reduction in AD. Extreme significance between AD vs. HC/MCI (*p* < 0.001); borderline significance between HC and MCI (*p* = 0.06). Panel **(1C)** (CSF p-tau181): HC (blue): Symmetric distribution clustered at low values. MCI (green): Right-shifted distribution with tail extending into AD range. AD (red): Pronounced rightward displacement with broad density, indicative of p-tau heterogeneity. Significant progression across groups (AD>MCI > HC; *p* < 0.001), supporting incremental tau pathology. Panel **(1D)** (CSF t-tau): HC (blue): Narrow low-value distribution. MCI (green): Right-shifted dispersion partially overlapping AD. AD (red): Distinct rightward shift with wide density. Hierarchical elevation (AD>MCI > HC; *p* < 0.001), commensurate with neurodegeneration severity. Panel **(2B)** (Left ALPS Index): HC (blue): Symmetric high-value clustering. MCI (green): Mild leftward shift with HC overlap. AD (red): Significant leftward displacement with narrow distribution with reduced density, suggesting functional impairment. Extreme AD vs. HC/MCI differences (*p* < 0.001); HC-MCI equivalence (*p* = 0.599), confirming ALPS disruption as AD-specific. Panel **(2C)** (Right ALPS Index): Parallel to left ALPS pattern with AD vs. HC. MCI fully overlapped HC. Consistent bilateral impairment (AD vs. HC/MCI *p* < 0.001; HC-MCI *p* = 0.722). Panel **(2D)** (Mean ALPS Index): AD (red): Narrow low-value clustering. HC (blue; peak ~1.31) and MCI exhibited overlapping high-value distributions. AD significantly lower than HC/MCI (*p* < 0.001) without HC-MCI difference (*p* = 0.608), supporting its role as late-stage AD biomarker. Panel **(3A)** (Left Hippocampal FA): HC (blue): High FA with narrow density. MCI/AD (green/red): Complete overlap with left-shifted distributions. MCI/AD vs. HC (*p* < 0.001); MCI-AD equivalence (*p* = 0.969), indicating stable microstructural damage at MCI stage. Panel **(3B)** (Right Hippocampal FA): Bilateral symmetry in damage patterns. MCI/AD vs. HC (*p* < 0.001); MCI-AD equivalence (*p* = 0.903), reinforcing early bilateral involvement. Panel **(3C)** (Left Hippocampal MD): HC (blue): Low MD with tight clustering. MCI (green): Right-shifted approaching AD range. AD (red): Extended rightward displacement with broad dispersion. Progressive elevation (AD>MCI > HC; *p* < 0.001) with non-significant MCI-AD difference (*p* = 0.067), suggesting slower left-sided deterioration. Panel **(3D)** (Right Hippocampal MD): AD (red): Pronounced right shift with wide dispersion. MCI (green): Intermediate partially overlapping AD. HC (blue): Low baseline. Significant MCI-AD progression (*p* = 0.014), demonstrating continued right hippocampal degeneration.

**Figure 5:**
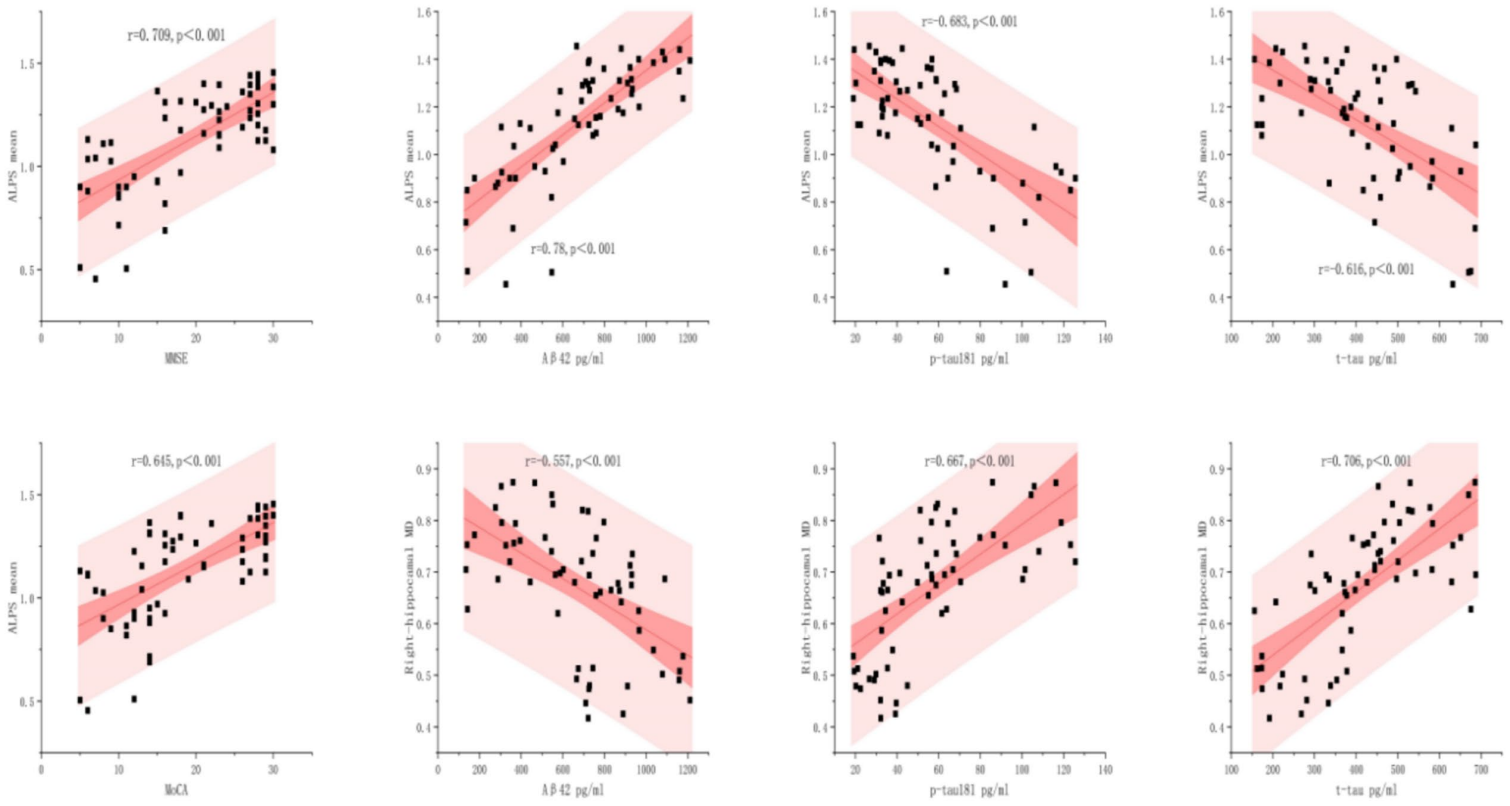
Relationships between biomarkers with ALPS mean and right-hippocampal MD. The scatterplots illustrate the linear associations between four clinical biomarkers with ALPS Mean and Right-hippocampal MD, with 95% confidence bands (dark pink) and prediction bands (light pink). Statistical significance is marked by *p* < 0.001.

**Figure 6:**
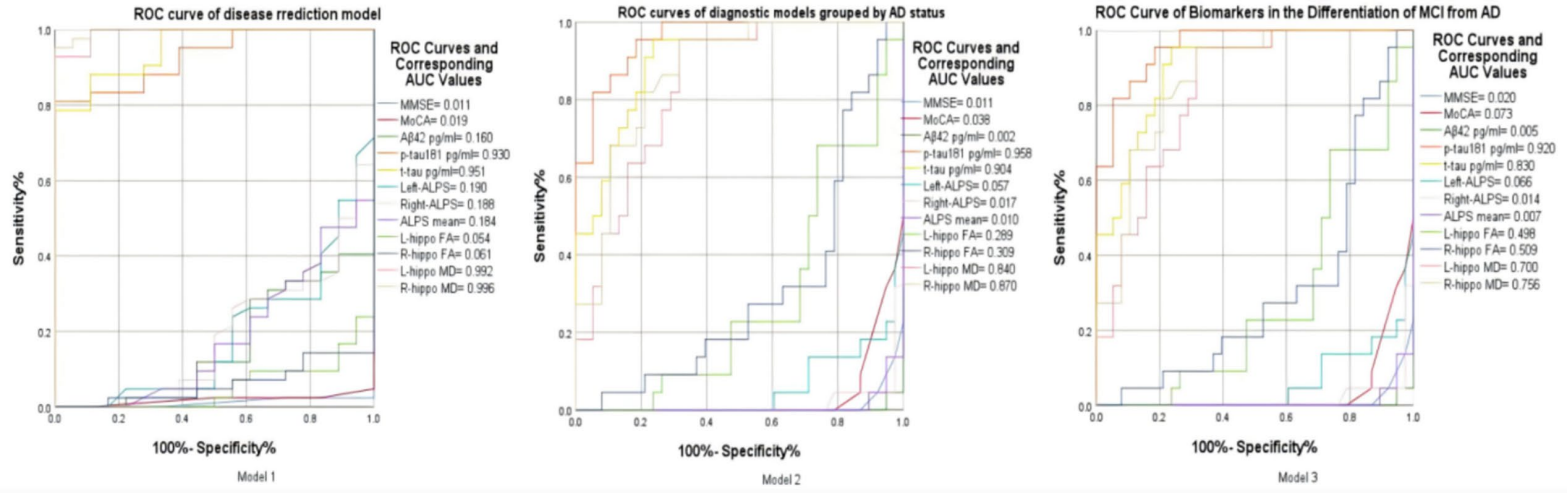
Model 1: ROC curve of key indicators for disease prediction based on grouping of diseased and non-diseased groups. Model 2: ROC curve of key biomarkers in the differentiation between AD and Non-AD. Model 3: ROC curve of imaging and protein indicators for the differentiation between MCI and AD.

Violin plots (Figure 4), scatterplots (Figure 5), and ROC curves (Figure 6) were generated using OriginPro 2024 (OriginLab, USA).

### 2.8 Diagnostic model construction

Three ROC models were designed: Model 1: Diseased (MCI + AD) vs. Non-diseased (HC); Model 2: AD vs. Non-AD (HC + MCI); Model 3: MCI vs. AD. Input features included right/left hippocampal MD/FA, CSF Aβ42/p-tau181/t-tau, and ALPS index. Binary logistic regression with leave-one-out cross-validation was applied.

## 3 Results

Key findings revealed three progressive patterns: (1) DTI-ALPS index significantly declined in AD versus HC/MCI (*p* < 0.001); (2) Hippocampal FA reduction plateaued at MCI stage while right MD showed continued progression to AD; (3) CSF biomarkers exhibited hierarchical amyloid depletion (Aβ42↓) and tau accumulation (t-tau↑). Multimodal correlations (e.g., Aβ42-ALPS: *r* = 0.78) and ROC analyses further validated clinical utility.

### 3.1 Statistical results of basic and clinical data

The study compares Healthy Controls (HC), Mild Cognitive Impairment (MCI), and Alzheimer’s Disease (AD) groups across demographic, cognitive, CSF biomarker, and MRI measures. Key findings are as follows:

#### 3.1.1 Demographics (sex, age, education)

No significant differences between groups (*p* > 0.05), ensuring observed changes are disease-related.

#### 3.1.2 Cognitive scores (MMSE and MoCA)

Significant decline from HC > MCI > AD (*p* < 0.001 for all comparisons), reflecting progressive cognitive impairment.

#### 3.1.3 CSF biomarkers: Aβ42

Lower in AD vs. HC/MCI (*p* < 0.001), with marginal HC vs. MCI (*p* = 0.06), suggesting Aβ42 drops markedly in AD. p-tau181 and t-tau: Increase from HC < MCI < AD (*p* < 0.001 for all comparisons except HC vs. MCI p-tau181: *p* = 0.006), indicating tau pathology progression.

#### 3.1.4 MRI measurements: ALPS index

Significant reduction in AD vs. HC/MCI (left, right, mean; *p* < 0.001), but no HC vs. MCI difference (p > 0.05). Suggests a novel marker for distinguishing AD from HC/MCI. Hippocampal FA: Lower in MCI/AD vs. HC (*p* < 0.001), but no MCI vs. AD difference (*p* > 0.90), indicating early damage in MCI that plateaus in AD. Hippocampal MD- Left MD: Higher in MCI/AD vs. HC (*p* < 0.001), with marginal MCI vs. AD increase (*p* = 0.067). Right MD: Significant MCI vs. AD difference (*p* = 0.014), suggesting asymmetric progression. While FA/MD changes occurred early in MCI, they plateaued in AD, except right MD, which progresses significantly. The combination of ALPS index and CSF biomarkers significantly improved diagnostic accuracy, while hippocampal diffusivity reflects early neurodegeneration in MCI.

### 3.2 Comprehensive visualization of biomarker distributions across HC, MCI, and AD groups

Figure 4 presents a comprehensive visualization of biomarker distributions across healthy controls (HC), mild cognitive impairment (MCI), and Alzheimer’s disease (AD) groups using a 4 × 3 grid of half violin plots. Each plot displays the distribution of key biomarkers, including cognitive scores (MMSE, MOCA), cerebrospinal fluid (CSF) biomarkers (Aβ42, p-tau181, t-tau), and magnetic resonance imaging (MRI) metrics (ALPS index, hippocampal fractional anisotropy (FA), and mean diffusivity (MD)). The half violin plots provide a detailed view of the median, quartiles, and density estimates, allowing for a nuanced comparison of biomarker distributions among the three groups. This visualization highlights the distinct biomarker trajectories associated with the progression of Alzheimer’s disease, emphasizing the utility of multimodal biomarkers in identifying and staging AD.

### 3.3 Correlation analysis

As shown in Figure 5, ALPS mean levels exhibited significant correlations with all biomarkers analyzed (*p* < 0.001). The strongest association was observed between A*β*42 and ALPS mean (*r* = 0.78), followed by MMSE (*r* = 0.709) and MoCA (*r* = 0.645). In contrast, p-tau181 and t-tau demonstrated inverse relationships (*r* = −0.683 and *r* = −0.616, respectively), these findings demonstrate distinct pathophysiological roles of amyloid-β and tau proteins in neurodegenerative pathology.

The right hippocampal MD demonstrated disease-stage dependent associations: strongly correlated with t-tau (*r* = 0.706, *p* < 0.001) and p-tau181 (*r* = 0.667, *p* < 0.001), but inversely associated with Aβ42 (*r* = −0.557, *p* < 0.001). Conversely, strong positive associations were found for p-tau (*r* = 0.667, *p* < 0.001) and t-tau (*r* = 0.706, *p* < 0.001).

### 3.4 Multiple ROC curves in disease diagnosis and differential diagnosis

In the ROC analysis of *Model 1*, which aims to distinguish between the diseased and non - diseased groups, the ROC curves of right hippocampal MD (AUC = 0.996), left hippocampal MD (AUC = 0.992), T-tau protein (AUC = 0.951), and P-tau protein (AUC = 0.930) are prominently located in the upper–left corner of the ROC space. This indicates their excellent discriminatory ability, with high sensitivity to correctly identify the diseased population and low false - positive rates to minimize misclassification of the non - diseased. In contrast, other variables’ ROC curves in the lower–right corner suggest poor performance in differentiating the two groups. These findings highlight the potential value of the former biomarkers in disease prediction models for this binary classification task (Figure 6).

For *Model 2*, which focuses on differentiating the AD group from the non–AD group, the ROC curves of P-tau 181(AUC = 0.958), T-tau (AUC = 0.904), right hippocampal MD (AUC = 0.870), and left hippocampal MD (AUC = 0.840) are positioned in the upper–left corner of the ROC space. This placement implies their strong diagnostic utility. They are characterized by high true - positive rates, enabling accurate detection of AD cases, and low false–positive rates, reducing the misdiagnosis of non–AD individuals as AD patients. In contrast, other curves in the ROC plot show relatively poor performance in this AD-non-AD discrimination. Overall, these four factors emerge as crucial indicators for accurate AD diagnosis within this model.

In *Model 3* for differentiating between MCI and AD, several metrics, including p – tau (AUC = 0.920), T-tau (AUC = 0.830), right hippocampal MD (AUC = 0.756), left hippocampal MD (AUC = 0.700) and right hippocampal FA (AUC = 0.509), are predominantly located towards the upper - left region of the ROC space. This distribution indicates their overall good discriminatory power in distinguishing MCI from AD. These metrics generally exhibit relatively high true positive rates (sensitivity), which means they can effectively identify a significant proportion of AD cases. Meanwhile, they maintain relatively low false positive rates (100%- specificity%), reducing the misclassification of MCI patients as AD patients. However, not all values are perfectly positioned in the extreme upper–left corner. There are some deviations, suggesting that while these metrics are useful, there is still room for improvement in their diagnostic accuracy. Overall, these metrics, on the whole, show promise in differentiating between MCI and AD, and further exploration of their combined application may lead to more accurate diagnostic models.

## 4 Discussion

This multimodal study elucidates the spatiotemporal dynamics of Alzheimer’s continuum through integrated assessment of perivascular structural integrity (via DTI-ALPS index), hippocampal microstructure, and CSF biomarkers. Three principal discoveries emerge: (1) DTI-ALPS index effectively discriminates AD from preclinical stages, while bilateral hippocampal fractional anisotropy (FA) reduction identifies early microstructural damage in MCI; (2) Right-dominant hippocampal mean diffusivity (MD) elevation reflects asymmetric neurodegeneration; (3) Multimodal biomarker correlations (Aβ42-ALPS: *r* = 0.78; t-tau-MD: *r* = 0.706) establish complementary roles of perivascular clearance and axonal degeneration in disease stratification. The strong inverse correlation between ALPS index and CSF p-tau181 (*r* = −0.683, *p* < 0.001) suggests impaired perivascular clearance potentiates tau accumulation. This aligns with glymphatic dysfunction models wherein AQP4 depolarization reduces tau efflux along perivascular channels. Notably, right-lateralized hippocampal MD progression (ΔMCI→AD = +0.06 × 10^−3^ mm^2^/s, *p* = 0.014) may reflect asymmetric default mode network (DMN) vulnerability (Li et al., 2013). Tau-PET studies indicate right precuneus/posterior cingulate hypometabolism precedes left hemisphere involvement in amnestic MCI (Ossenkoppele et al., 2016), potentially explaining the right > left MD trajectory observed here (Lavrova et al., 2025).

Our DTI-ALPS findings extend prior reports on perivascular structural alterations in AD (Taoka et al., 2017), demonstrating its specificity for distinguishing AD from MCI (*p* < 0.001). The preserved perivascular integrity in MCI (ALPS index = 1.26 ± 0.09 vs. AD = 0.87 ± 0.19) contrasts with early hippocampal FA reduction (0.57 ± 0.11 vs. HC = 0.82 ± 0.07), suggesting temporal decoupling between hippocampal degeneration and perivascular impairment. This supports a sequential model: initial hippocampal microstructural disruption (FA/MD changes) may prime Aβ deposition (Harrison et al., 2020), while subsequent perivascular clearance failure (ALPS decline) accelerates tau-mediated network collapse (Nedergaard and Goldman, 2020). The right hippocampal MD predominance (*p* = 0.014,) aligns with tau-PET documented hemispheric vulnerability gradients (Ossenkoppele et al., 2016), possibly mediated by default mode network metabolic asymmetry (Li et al., 2017). The Aβ42-ALPS correlation (*r* = 0.78, *p* < 0.001) mechanistically links amyloid accumulation to perivascular drainage dysfunction. Preclinical studies confirm that perivascular basement membrane thickening impedes Aβ clearance through intramural periarterial pathways (Zhang et al., 2021). Furthermore, the strong inverse relationship between t-tau and p-tau181 with ALPS (*r* = −0.616 and *r* = −0.683, respectively) suggests that as tau pathology progresses, perivascular clearance mechanisms decline, potentially contributing to the accumulation of tau proteins within the brain. This finding is consistent with the proposed role of perivascular drainage in the clearance of tau from the brain (Wu et al., 2021). The observed temporal decoupling between hippocampal degeneration and perivascular impairment provides insights into the potential mechanisms underlying the progression of Alzheimer’s disease. The early involvement of the hippocampus, as evidenced by FA reduction, may represent an initial step in the disease process, with subsequent perivascular clearance failure accelerating the neurodegenerative cascade (Liu et al., 2021). This sequential model highlights the importance of considering both hippocampal microstructural changes and perivascular alterations in the understanding and treatment of Alzheimer’s disease.

Diffusion tensor imaging (DTI) is conventionally applied to white matter analysis, our detection of hippocampal fractional anisotropy (FA) and mean diffusivity (MD) alterations aligns with evidence that DTI sensitively captures gray matter microstructural changes in neurodegeneration (Zhang et al., 2014). Specifically, hippocampal MD elevation reflects neuronal loss and expansion of extracellular space (Li et al., 2013), while reduced FA correlates with underlying neuropathology (Snow et al., 2017). Future studies should integrate advanced gray matter-specific metrics (e.g., neurite orientation dispersion via NODDI) with tau-PET imaging to enhance the quantification of hippocampal dysfunction (Lavrova et al., 2025).

While this study presents promising advancements, it is essential to acknowledge its limitations. The relatively small sample size and lack of longitudinal follow-up may restrict the generalizability of the findings. While cross-sectional design limits causal inference, our proposed “FA → MD → ALPS” progression sequence warrants longitudinal verification. Single-center sampling (*N* = 60) necessitates validation in multiethnic cohorts, particularly given ethnic variability in perivascular anatomy. Future research should aim to address these limitations by incorporating larger, multi-center studies with extended follow-up periods. Manual ALPS quantification introduces measurement bias; emerging deep learning pipelines could enhance reproducibility. The DTI-ALPS index, though sensitive to perivascular changes, may conflate PVS enlargement with adjacent white matter lesions. Future studies could combine ALPS with dynamic contrast-enhanced MRI to directly measure glymphatic flow.

In conclusion, our multimodal study demonstrates the complementary roles of perivascular clearance and axonal degeneration in Alzheimer’s disease stratification. DTI-ALPS quantifies advanced-stage perivascular integrity loss, whereas right hippocampal MD tracks asymmetric neurodegeneration. The temporal dissociation between early hippocampal changes (MCI-specific) and late perivascular decline (AD-specific) suggests stage-dependent biomarker utility: FA/MD for early detection, ALPS for therapeutic monitoring, provides a comprehensive view of the spatiotemporal dynamics of Alzheimer’s continuum. The ROC curve analysis (Model 2 in Figure 6) highlights the superior diagnostic performance of right hippocampal MD and CSF t-tau in differentiating AD from non-AD groups, while the combination of hippocampal diffusivity metrics (FA/MD) and CSF p-tau181 provides enhanced accuracy in distinguishing MCI from AD. These findings have important implications for the development of targeted therapies and the improvement of diagnostic accuracy in Alzheimer’s disease.

## Data Availability

The raw data supporting the conclusions of this article will be made available by the authors, without undue reservation.
